# Urinary Ailments: Therapeutics

**Published:** 1907-11

**Authors:** W. C. Abbott

**Affiliations:** Chicago, Ill.


### Urinary Ailments: Therapeutics.

BY W. C. ABBOTT, M. D., CHICAGO, ILL.

Urinary retention from chill, subinflammatory, is relieved by aconitine a granule gr. 1-134 every one or two hours till the pulse softens or relief ensues.

Retention or incontinence, or both, in the aged is relieved by strychnine, any salt, gr. 1-134 to 1-30 every two to four hours until normal tone of pulse is attained.

Frequent and involuntary micturation and the dribbling of women are helped by small doses of cantharidin, gr. 1-5000 every hour till slight vesicle irritation.

The retention that occurs during the progress of spinal maladies is benefited by ext. cannabis indica, gr. 1-6 every one to three hours.

Sudden suppression from cold, damp, scarlatina, etc., is benefited by digitalin, gr. 1-67 every hour till the secretion is restored or pulse is tense enough.

Catarrhal irritation, retention or incontinence, irritable stricture or gleety debility, are relieved by barosmin, a grain every one to four hours.

For alkaline or phosphatic urine give acid benzoic, gr. 1-6 every hour, which is far better than larger and less frequent dosage.

For spasmodic strictures, and to prevent chill or fever after passing urethral instruments, give aconitine, gr. 1-134 every half hour to two hours.

For any catarrh of the urinary apparatus give cubebin in very small doses, repeated every hour or two and continued for rather long periods.

For alkaline urine give one of the mineral acids, preferably phosphoric, in small and oft-repeated doses.

Weight sensations in the perineum, dragging on the testicles, difficult or lagging urination and strangury, are relieved by senecin, a grain every two to four hours.

All catarrhal urinary maladies are best relieved and most surely cured by arbutin, gr. 1 daily in divided doses.

Even the terrible cystitides of gonorrheal infection are cured by arbutin, which may be required in doses up to 30 grains a day, continued for months.

Urinary inflammations and suppurations totally incurable by any other remedies are cured by arbutin, but it requires more patience than surgery.

Relaxed states of the urinary mucosa are remedied by berberine, which should be given in doses up to one to seven grains a day for one to three months.

Hemorrhagic urinary maladies are benefited by hydrastine, gr. 1-67 to 1-12 seven times a day; a slow-acting but sure hemostatic here.

All vesic irritations are relieved by hyoscine hydrobromate, gr. 1-1000 every half hour till effect or drowsiness supervenes.

Spasmodic affections of the urinary ways are most speedily relieved by atropine or hyoscyamine, gr. 1-500 every half hour till the mouth begins to feel dry.

Very small doses of rhus tox. stimulate the vitality of the urinary tissues and this may be needed to set up curative processes.

For the tendency to eject urine when coughing, sometimes pestering women, give atropine enough to decidedly dry the mouth, and rhus to irritative effect following.

The treatment of many vesic irritations should begin in the stomach, duodenum, liver or rectum; or with the diet and other personal habits.

When we think of the gonorrheal miseries we mistreated before we had arbutin, we haven't any conceit left in us.

There is no reason for relying so exclusively on arbutin as to neglect saturation with calyx sulphurate in gonorrheal maladies.

Cocaine and similar remedies applied to the surface of the glans decidedly relieve irritations of the bladder without the trouble and danger of irrigations.

Hematurias may require for permanent cure either erigeron or eucalyptus oil in doses of gr. 1-5 every four to eight hours—a powerful remedy for bad cases only.

Must we remark that the removal of any removable cause of irritation should precede any treatment directed against the irritation itself?

When we see a vesical irritation that operation and drainage failed to relieve get well under arbutin, we begin to doubt if there is no treatment except surgery.

---

Sudden marked rise of temperature a few days after an operation for appendicitis, especially if attended by chills, may mean thrombosis of the portal vein or multiple abscesses of the liver.—*American Journal of Surgery.*

